# Genome Sequence of *Escherichia coli* Strain LCT-EC52, Which Acquired Changes in Antibiotic Resistance Properties after the Shenzhou-VIII Mission

**DOI:** 10.1128/genomeA.00081-14

**Published:** 2014-03-06

**Authors:** Dong Zhang, De Chang, Xuelin Zhang, Yi Yu, Yinghua Guo, Junfeng Wang, Tianzhi Li, Guogang Xu, Wenkui Dai, Changting Liu

**Affiliations:** a Nanlou Respiratory Diseases Department, Chinese PLA General Hospital, Beijing, China; b BGI-Shenzhen, Shenzhen, China

## Abstract

*Escherichia coli* is a ubiquitous opportunistic pathogen that colonizes the lower intestines of humans and causes several diseases, such as septicemia, pneumonia, and urinary tract infections. Here, we present the draft genome sequence of *E. coli* strain LCT-EC52, which originated from *E. coli* strain CGMCC 1.2385 and acquired changes in antibiotic resistance following travel on the Shenzhou-VIII spacecraft.

## GENOME ANNOUNCEMENT

There are many species of bacteria found in the human body, most of which are normal flora. *Escherichia coli* is a Gram-negative rod-shaped bacterium that commonly resides in the lower intestines of humans and mammals. When the immune system is compromised, *E. coli* can cause various diseases, including bacteremia, septicemia, meningitis, urinary tract infection, cholecystitis, cholangitis, dysentery, and pneumonia. It is reported that space flight might decrease host immunity, which may increase the risk of contracting infectious diseases related to *E. coli* strains that are harbored in the human body during space travel (1, 2). Thus, it is necessary to investigate the effect of space travel on *E. coli*. The *E. coli* strain CGMCC 1.2385 was loaded on the Shenzhou-VIII spacecraft, which traveled into space for 17 days. *E. coli* strain LCT-EC52 was isolated and showed significantly increased resistance to ampicillin, cefazolin, ceftazidime, ceftriaxone, and azithromycin (3).

The nucleotides were fragmented to generate 500-bp and 6-kb fragment libraries. PCR was performed to amplify these 500-bp and 6-kb libraries for ~100× and ~50× genomic coverage, respectively. We performed paired-end sequencing of the libraries using an Illumina HiSeq 2000 according to the manufacturer’s instructions. Using SOAP*denovo* (version 1.6), 90-bp reads from the two libraries were assembled into 191 contigs according to methods published previously and were connected to 37 scaffolds using information from paired-end sequencing of the 6-kb library. The total assembled nucleotide count is 5,209,752 bp, including 106,472 bp of unknown bases (gaps). The *N*_50_ of the assembled scaffolds is 2,676,640 bp, and the G+C content is 50.37%. To predict coding sequences in the scaffolds, we used Glimmer version 3.0 to predict putative open reading frames. The resulting coding sequences (CDSs) were aligned to the NR, COG, and KEGG databases. We identified tandem repeat sequences using TRF version 4.04 and scattered repeat sequences using RepeatMasker version 3.2.9. rRNA and tRNA sequences were identified with RNAmmer and tRNAscan-SE 1.21, respectively. Virulence genes and antibiotic resistance genes were detected by aligning CDSs to VFDB and ARDB, respectively.

### Nucleotide sequence accession number.

This whole genome of *E. coli* LCT-EC52 has been deposited at DDBJ/EMBL/GenBank under the accession no. ANHT00000000. The versions described in this paper are the first versions.
